# Supplementary material for: Enhanced Recovery and Detection of Highly Infectious Animal Disease Viruses by Virus Capture Using Nanotrap® Microbiome A Particles
Source: Viruses. 2024 Oct 23;16(11):1657. doi: 10.3390/v16111657 (PMC11599081; doi:10.3390/v16111657)
Supplement: Supplementary file 1 [file viruses-16-01657-s001.zip › viruses-3250800-supplementary.pdf]

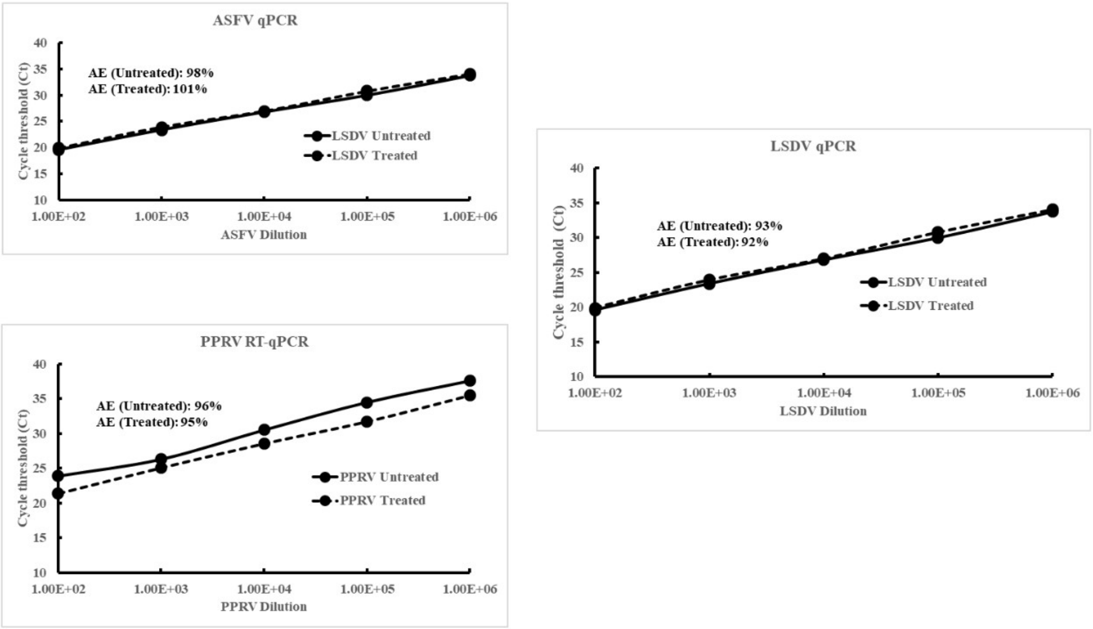

Supplemental Figure S1. Linear regression standard curves and amplification efficiencies (AE) of the viral DNA/RNA extracted from serial dilutions of the viruses (LSDV, PPRV or ASFV) with (+NMAPS) or without (-NMAPs) virus capture. Viruses were serially (10-fold) diluted in PBS and used as the starting material. For untreated samples, 200 ml of virus from each dilution was extracted directly and analyzed by qPCR/RT-qPCR. For treated (+NMAPs) samples, the same amount of virus (200 µl) from each dilution was further diluted 1:50 (10 ml) in PBS and then subjected to virus capture using NMAPs. The NMAPs and the captured viruses were clarified on a magnetic stand, reconstituted in PBS (200 ml), extracted (viral DNA/RNA), and analyzed by virus-specific qPCR/RT-qPCR. A, standard curve of ASFV DNA extracted from serial dilutions; B, standard curve of PPRV RNA extracted from serial dilutions; and C, standard curve of LSDV DNA extracted from serial dilutions. The inset shows amplification efficiency (AE) calculated from the standard curves. Each data point (Ct values) represents an average of 3 replicates with a standard deviation of “mean ± 0.155”.

Table S1. Diagnostic sensitivity of ASFV qPCR with DNA extracted from specimens of experimentally infected animals after virus capture using NMAPs.

Virus	Host	# Animals	Dpi	Specimen	Animal ID	Cycle threshold (qPCR)	
						Untreated* (-NMAPs)	Treated# (+NMAPs)
ASFV	Swine	20	7	EWB	52468	17.939	17.111
					52469	17.481	17.084
					52470	16.115	16.124
					52471	16.855	16.243
					52.792	18.479	18.120
					52793	20.388	20.072
					52795	19.375	19.165
					52796	17.905	17.649
					53465	28.951	28.541
					53466	20.162	19.326
					52467	22.593	21.554
					53468	20.900	20.495

					53469	19.637	19.100
					53470	16.111	15.224
					53471	16.855	16.243
					53838	30.569	30.211
					53839	21.758	21.123
					53841	17.283	17.032
					53842	18.500	17.632
					53843	16.752	16.068

*Untreated: 100 µl of EWB was diluted 1:1 in PBS (100 µl) and then extracted and analyzed by ASFV specific qPCR.

#Treated: 100 µl of EWB was diluted in PBS (2 ml final volume) and then subjected to virus capture using NMAPs; the NMAPs and the captured viruses were extracted as described in the legends to Table 5 and analyzed by ASFV specific qPCR. .

Table S2. Diagnostic sensitivity of PPRV RT-qPCR with RNA extracted from specimens of experimentally infected animals with (treated) or without (untreated) virus capture using NMAPs.

Virus	Host	# Animals	Dpi	Specimen	Animal ID	Cycle threshold (qPCR)	
						Untreated* (-NMAPs)	Treated# (+NMAPs)
PPRV	Goat	8	8	EWB	23-1	29.814	29.156
					23-2	28.097	28.898
					23-3	30.107	27.365
					23-4	28.025	27.430
					23-13	32.076	30.060
					23-14	28.198	27.535
					23-15	33.507	33.197
					23-16	31.424	31.160
				Nasal swab	23-1	20.993	19.201
					23-2	28.477	26.065
					23-3	29.109	27.197
					23-4	20.364	18.708
					23-13	24.098	25.993
					23-14	26.279	27.479
					23-15	28.097	30.306
					23-16	26.814	24.128
				Oral	23-13	25.407	27.432
					23-14	27.801	29.069
					23-15	31.847	30.905
					23-16	28.884	27.814
				Conjunctival swab	23-1	21.767	19.994
					23-2	25.942	25.302
					23-3	29.991	26.679
					23-4	26.068	25.326

					23-13	23.608	27.526
					23-14	26.845	26.492
					23-15	22.841	23.954
					23-16	22.502	23.016

*Untreated: 200 µl of swabs or 200 µl of 1:1 diluted (in PBS) EWB were extracted and analyzed by PPRV specific RT-qPCR.

#Untreated: 200 µl of swabs or 100 µl of EWB diluted in PBS (2 ml final volume) and then subjected to virus capture using NMAPs; the NMAPs and the captured viruses were extracted as described in the legends to Table 5 and analyzed by PPRV specific RT-qPCR.

Table S3. Diagnostic sensitivity of SPPV qPCR with DNA extracted from specimens of experimentally infected animals after virus capture using NMAPs.

Virus	Host	# Animals	Dpi	Specimen	Animal ID	Cycle threshold (qPCR)	
						Untreated* (-NMAPs)	Treated# (+NMAPs)
SPPV	Sheep	6	10	Oral swab	23-20	26.344	31.046
					23-21	28.602	29.857
					23-22	33.764	32.054
					23-28	30.836	32.176
					23-29	33.162	32.513
					23-30	32.458	32.871
				Nasal swab	23-20	29.088	22.960
					23.21	25.898	26.889
					23-22	37.022	37.407
					23-28	24.412	25.454
					23-29	29.240	31.502
					23-30	29.284	31.072
				Conjunctival; swab	23-20	27.779	29.219
					23-21	27.600	29.739
					23-22	35.546	35.869
					23-28	18.085	19.427
					23-29	26.118	28.356
					23-30	28.167	28.871
				EWB	23-20	24.550	23.989
					23-21	27.415	27.479
					23-22	23.393	21.898
					23-28	27.8097	27.066
					23-29	23.406	23.050
					23-30	21.470	20.604

*Untreated: 200 µl of swabs or 200 µl of 1:1 diluted (in PBS) EWB were extracted and analyzed by SPPV specific qPCR.

#Untreated: 200 µl of swabs or 100 µl of EWB diluted in PBS (2 ml final volume) and then subjected to virus capture using NMAPs; the NMAPs and the captured viruses were extracted as described in the legends to Table 5 and analyzed by SPPV specific qPCR.

Table S4. Diagnostic sensitivity of ASFV qPCR with DNA extracted from EWB of naturally infected swine after virus capture using NMAPs.

Virus	Host	# Animals	Specimen	Animal ID	Cycle threshold (qPCR)	
					Untreated* (-NMAPs)	Treated# (+NMAPs)
ASFV	Swine	20	EWB	14	18.06	17.470
				27	19.526	19.056
				29	19.423	19.154
				30	18.722	18.372
				32	22.583	20,540
				36	22.432	21.468
				37	20.704	19.779
				39	18.155	17.823
				41	18.618	18.214
				42	22.822	22.890
				43	36.399	35.919
				44	22.325	22.221
				45	18.859	17.599
				46	20.383	20.061
				52	19.443	19.137
				53	19.309	18.443
				54	18.489	18.786
				58	17.624	17.362
				59	22.920	22.330
				60	20.967	20.260

*Untreated: 100 µl of EWB was diluted 1:1 in PBS (100 µl) and then extracted and analyzed by ASFV specific qPCR.

#Treated: 100 µl of EWB was diluted in PBS (2 ml final volume) and then subjected to virus capture using NMAPs; the NMAPs and the captured viruses were extracted as described in the legends to Table 5 and analyzed by ASFV specific qPCR.
